# Epigenetic mechanisms of lung carcinogenesis involve differentially methylated CpG sites beyond those associated with smoking

**DOI:** 10.1007/s10654-022-00877-2

**Published:** 2022-05-20

**Authors:** Dusan Petrovic, Barbara Bodinier, Sonia Dagnino, Matthew Whitaker, Maryam Karimi, Gianluca Campanella, Therese Haugdahl Nøst, Silvia Polidoro, Domenico Palli, Vittorio Krogh, Rosario Tumino, Carlotta Sacerdote, Salvatore Panico, Eiliv Lund, Pierre-Antoine Dugué, Graham G. Giles, Gianluca Severi, Melissa Southey, Paolo Vineis, Silvia Stringhini, Murielle Bochud, Torkjel M. Sandanger, Roel C. H. Vermeulen, Florence Guida, Marc Chadeau-Hyam

**Affiliations:** 1 Department of Epidemiology and Biostatistics, MRC Centre for Environment and Health, School of Public Health, Imperial College London, St Mary’s Campus, Norfolk Place, London, W2 1PG UK; 2 Department of Epidemiology and Health Systems (DESS), University Centre for General Medicine and Public Health (UNISANTE), Lausanne, Switzerland; 3 Department and Division of Primary Care Medicine, University Hospital of Geneva, Geneva, Switzerland; 4 Bureau de Biostatistique et d’Épidémiologie, Institut Gustave Roussy, Université Paris-Saclay, Villejuif, France; 5 Oncostat U1018, Inserm, Équipe Labellisée Ligue Contre Le Cancer, Université Paris-Saclay, Villejuif, France; 6 Department of Community Medicine, UiT The Arctic University of Norway, Tromsø, Norway; 7 Italian Institute for Genomic Medicine, Turin, Italy; 8 Molecular and Nutritional Epidemiology Unit, Cancer Research and Prevention Institute-ISPO, Florence, Italy; 9 Epidemiology and Prevention Unit, Fondazione IRCCS Istituto Nazionale dei Tumori, Milan, Italy; 10 Hyblean Association for Epidemiological Research, AIRE- ONLUS, Ragusa, Italy; 11 Unit of Cancer Epidemiology Città Della Salute e della Scienza University-Hospital, Via Santena 7, 10126 Turin, Italy; 12 Department of Clinical Medicine and Surgery, Federico II University, Naples, Italy; 13 The Norwegian Cancer Registry, Oslo, Norway; 14 Cancer Epidemiology Division, Cancer Council Victoria, Melbourne, Australia; 15 Centre for Epidemiology and Biostatistics, Melbourne School of Population and Global Health, The University of Melbourne, Melbourne, Australia; 16 Precision Medicine, School of Clinical Sciences at Monash Health, Monash University, Clayton, Australia; 17 Centre for Research in Epidemiology and Population Health, Inserm (Institut National de La Sante Et de a Recherche Medicale), Villejuif, France; 18 Department of Clinical Pathology, Melbourne Medical School, The University of Melbourne, Melbourne, Australia; 19 Institute for Risk Assessment Sciences, Utrecht University, Utrecht, The Netherlands; 20 Julius Centre for Health Sciences and Primary Care, University Medical Centre, Utrecht, Utrecht, The Netherlands; 21 Group of Genetic Epidemiology, International Agency for Research on Cancer (IARC) – World Health Organization (WHO), Lyon, France

**Keywords:** Lung cancer, Smoking, DNA methylation, Variable selection, Partial correlation network

## Abstract

Smoking-related epigenetic changes have been linked to lung cancer, but the contribution of epigenetic alterations unrelated to smoking remains unclear. We sought for a sparse set of CpG sites predicting lung cancer and explored the role of smoking in these associations. We analysed CpGs in relation to lung cancer in participants from two nested case–control studies, using (LASSO)-penalised regression. We accounted for the effects of smoking using known smoking-related CpGs, and through conditional-independence network. We identified 29 CpGs (8 smoking-related, 21 smoking-unrelated) associated with lung cancer. Models additionally adjusted for Comprehensive Smoking Index-(CSI) selected 1 smoking-related and 49 smoking-unrelated CpGs. Selected CpGs yielded excellent discriminatory performances, outperforming information provided by CSI only. Of the 8 selected smoking-related CpGs, two captured lung cancer-relevant effects of smoking that were missed by CSI. Further, the 50 CpGs identified in the CSI-adjusted model complementarily explained lung cancer risk. These markers may provide further insight into lung cancer carcinogenesis and help improving early identification of high-risk patients.

**Supplementary Information:**

The online version contains supplementary material available at 10.1007/s10654-022-00877-2.

## Background

Although tobacco smoking has been identified as the leading cause of lung cancer since the early 1950’s, and despite considerable progress in the diagnosis and treatment of the disease, lung cancer is still the leading cause of cancer-related deaths worldwide. Lung cancer survival remains dismal (five-year survival rate ranging from 10 to 20%) mainly due to patients being diagnosed at later stages of the disease [1–4]. The lack of effective early diagnostic tools is in part resulting from the incomplete understanding of the molecular mechanisms involved in the carcinogenesis process. Moreover, in populations where the prevalence of smoking is low, an increasing proportion of lung cancer occurs in never-smokers and former smokers [1] highlighting a pressing need for identifying specific molecular mechanisms involved in lung carcinogenesis unrelated to smoking.

While the smoking-induced genetic changes affecting oncogenes and tumour suppressor genes have now been well-established [5], recent findings have shown that tobacco use also leads to *epigenetic* modifications through DNA methylation at Cytosine-phosphate-Guanine (CpG) sites, potentially resulting in modified gene expression [2, 5]. Previous studies have shown that the lung epithelium, and other tissues such as blood, buccal cells, and sputum, present relatively conserved methylation profiles in relation to smoking and/or lung cancer [6, 7]. Such epigenetic signatures include methylation changes in CpG sites related to inflammation, detoxification of xenobiotics, or cell proliferation [2, 6, 8–10].

Several studies have investigated the role and potentially mediating molecular pathways affected by smoking-related epigenetic changes in relation to lung carcinogenesis [11–15], but so far, the potential contribution of smoking-independent epigenetic modifications to lung cancer development remains understudied.

In the present study, we used full-resolution DNA methylation data from two European cohorts, the Italian component of the European Prospective Investigation into Cancer and Nutrition (EPIC-Italy) and the Norwegian Women and Cancer Study cohort (NOWAC). We used (LASSO) penalised logistic regression models calibrated via stability selection to identify robust sets CpG sites that are jointly explanatory of future risk of lung cancer. We subsequently investigated to what extent these associations were driven by exposure to tobacco smoke, and further assessed the performance of selected (sets of) CpG sites in predicting lung cancer in comparison to established metrics of tobacco smoke exposure. Finally, we investigated the complex correlation across selected CpG sites and their relationship with smoking via conditional independence network inference.

## Methods

### Study population

The present work uses data from two European population-based prospective cohorts: the Italian component of the European Prospective Investigation into Cancer and Nutrition (EPIC-Italy, N = 697 men and women aged 35–72) and the Norwegian Women and Cancer Study Cohort (NOWAC, N = 442 women aged 35–65) [6].

EPIC-Italy included over 47,000 participants between 1993 and 1998, who provided anthropometric measurements, blood samples, and information on medical history and lifestyle factors collected through a self-administered questionnaire [6, 11]. Upon sampling, blood was transported to local laboratories and prepared for DNA extraction according to standard laboratory protocols [6, 11]. Within EPIC-Italy, we used data from a lung cancer nested case–control (CC) study including 192 incident lung cancer cases and 192 healthy controls matched to cases by sex, date of birth, date of inclusion, and study centre. Additionally, we included 322 healthy control individuals from a breast and colon cancer study nested within EPIC-Italy [11] (Flow chart: Supplementary Fig. 1).

The NOWAC study recruited over 172,000 participants between 1991 and 2007 and collected anthropometric measurements, and self-reported information on medical history and lifestyle factors [16]. In a subset of (N = 50,000) participants, recruited between 2003 and 2006, a blood sample was also available. Upon collection, blood samples were sent out to the Department of Community Medicine at the University of Tromsø and subsequently prepared for DNA extraction according to previously described laboratory protocols [6, 17]. During the follow-up and to the end of 2011, 132 lung cancer cases with blood samples were identified and were used for the DNA methylation profiling [11]. For each case, one control with an available blood sample was selected and matched on time since blood sampling and year of birth. We also included data from 190 healthy control individuals from a breast and colon cancer study nested within NOWAC (Supplementary Fig. 1) [11].

We excluded seven and four lung cancer cases from EPIC-Italy and NOWAC, respectively, as well as two and eight controls, due to blood samples not passing the quality control checks, or to missing data for one or more covariates. This left 313 lung cancer cases (185 in EPIC-Italy, and 128 in NOWAC) and 826 controls (512 in EPIC-Italy, and 314 in NOWAC) for statistical analyses (Supplementary Fig. 1). Both EPIC-Italy and NOWAC studies were approved by relevant international, national, and local ethics committees and all participants provided signed informed consent.

### CpG methylation measurement and data pre-processing

For both cohorts, epigenome-wide analyses were carried out from whole blood cells DNA using the IlluminaInfiniumHumanMethylation450 array, with all laboratory procedures performed at the Human Genetics Foundation (Turin, Italy) according to the manufacturer’s protocols [6]. In EPIC-Italy, data pre-processing was performed using in-house scripts as previously described [6]. Briefly, for each sample and each probe, measurements were set to missing if obtained by averaging intensities over less than three beads, or if averaged intensities were below detection thresholds estimated from negative control probes. Background subtraction and dye bias correction (probes using Infinium II design) were also performed. This procedure resulted in a subset of 473,929 CpG sites, of which probes detected in < 20% of the samples were excluded, yielding 465,886 CpG markers in the lung cancer nested study, and 443,150 in the breast and colon cancer controls samples (Supplementary Fig. 1). The same pre-processing procedure was performed for NOWAC, yielding 485,512 CpGs suitable for analyses. The final, merged study sample included 443,150 CpGs available in both cohorts, for a total of 1139 participants.

Further data processing included imputation of missing methylation values according to the k-nearest neighbour procedure (k = 10), followed by the M-transformation of imputed CpG data, expressed as log_2_-transformed ratios of intensities arising from methylated cytosines over those arising from unmethylated cytosines.

To account for technically induced noise, we adopted a two stage ‘denoising’ strategy fitting a linear mixed model for the methylation level at each CpG site (as outcome variable) as a function of age at blood collection, sex, BMI, and case control status as fixed effect (predictors), and including the technical confounders: chip ID (177 modalities), position of the sample on the chip (12 modalities), and recruitment centre (six modalities) as random intercepts. Denoised methylation levels were then obtained by subtracting for the observed levels the estimated random effects [18, 19].

### Statistical analyses

#### CpG site classification

We categorized the selected 443,150 CpG sites into two groups based on their reported association with smoking, using data from the largest meta-analysis investigating the effects of smoking on epigenome-wide CpG markers [20]. We define ‘smoking-related’ CpG sites as those (N = 2623) found associated with smoking in that meta-analysis at a Bonferroni-corrected significance level ensuring a control of the Family Wise Error Rate below 0.05. Conversely, we define ‘smoking-unrelated’ CpG sites (N = 440,527) as those not associated with any of the smoking metrics.

#### Multivariate regression

Penalised logistic regression using Least Absolute Shrinkage and Selection Operator regularization (LASSO) was used to perform variable selection and identify a sparse set of CpG sites complementarily contributing to the risk of lung cancer. Models were adjusted for sex and age at blood collection *(base model)*. To account for potential confounding by exposure to tobacco smoking, we also considered models further adjusted for the Comprehensive Smoking Index (CSI), a continuous score accounting for the duration and the intensity of smoking across the life-course (*CSI-adjusted model*) [21]. Adjustment was achieved by not applying any penalisation to the regression coefficients of the adjustment variables [22, 23]. To ensure reproducibility of the findings, LASSO regression was calibrated via stability by means of resampling [24]. Selection proportions for each predictor were computed over 1000 subsamples of 80% of the study participants. The proportion of cases and controls in each subsample was controlled to be representative of that in the full population. Stably selected CpG sites were defined as those with selection proportions, computed over models fitted with a given penalty parameter, above a threshold. The penalty parameter and threshold in selection proportions are jointly calibrated by maximising a stability score derived from the likelihood of uniform (i.e. uninformative) feature selection [25]. The average beta-coefficients, conditionally on selection, estimated over the 1000 LASSO models with calibrated penalty are reported. To assess potential confounding by blood cell composition differentials, we ran our variable selection model adjusting (i.e. by non penalising) for estimated blood cell type proportions. We estimated blood cell type composition from the sentinel CpG sites proposed by Houseman [26] and of the six estimated proportions we adjusted for proportions of Monocytes, B cells, CD4 + T cells, Natural Killers, CD8 + T cells, and Neutrophil. We report and compare the recalibrated effect size estimates for the model without and with adjustment for blood cell composition.

#### ROC analysis

Logistic regression models including the stably selected CpG sites were recalibrated on 1000 training sets (80% of the samples), and the discriminatory ability of each model was assessed in the out-of-bag test set (remaining 20% of the samples) by estimating the sensitivity, specificity, and Area under the Receiver Operating Characteristic (ROC) curve. Performances are reported in terms of average, 5th and 95th percentiles for these metrics computed across the 1000 test sets. In addition, to quantify the amount of information brought about by each of the stably selected CpG sites, a series of models sequentially adding the CpG sites by order of selection proportion were evaluated and their AUC was reported.

#### Conditional independence networks

To better characterize DNA methylation changes associated with the future risk of lung cancer, we constructed a conditional independence network of lung cancer related CpG sites from the stability selection LASSO and also included CSI as a node in the network. The partial correlation network was estimated using stability selection applied to the graphical LASSO [24]. Selection proportions of the edges were estimated on (N = 1000) subsamples of 50% of the population and our calibration jointly defined the penalty of the graphical LASSO and the threshold in selection proportion for an edge to be considered stable by maximizing the stability score, while ensuring that the expected number of False Positive selected edges is below 10 [25].

All statistical analyses were carried out using the R statistical software (version 4.0.3) using glmnet package and in-house script for stability selection and conditional independence networks. These available upon request to the corresponding author.

## Results

### Descriptive analyses

The characteristics of the study population are presented in Table 1. In EPIC-Italy, the mean age of participants at blood sampling was 54 years for controls, and 54.5 for lung cancer cases, whereas lung cancer cases were older in NOWAC (56 vs. 51.1 years). In both EPIC-Italy and NOWAC, there was a higher proportion of current and former smokers in lung cancer cases, as well as a higher smoking duration, smoking intensity and CSI.

**Table 1:** Characteristics of study participants stratified by cohort and future lung cancer status. The mean (standard deviation) and counts (percentage) are reported for continuous and categorical variables respectively

	EPIC-Italy		NOWAC		Full population
	Controls (N = 512)	Cases (N = 185)	Controls (N = 314)	Cases (N = 128)	
Sex (women)	331 (65%)	81 (44%)	314 (100%)	128 (100%)	854 (75%)
Age (years)	54 (6.8)	54.5 (6.3)	51.1 (6.9)	56 (4.2)	53.5 (6.7)
*Smoking status*					
Never	257 (50%)	26 (14%)	136 (43%)	14 (11%)	433 (38%)
Former	143 (28%)	59 (32%)	97 (31%)	34 (27%)	333 (29%)
Current	112 (22%)	100 (54%)	81 (26%)	80 (62%)	373 (33%)
Smoking duration (years)	12.1 (14.2)	27.3 (14.3)	15.9 (16.5)	31.6 (14.8)	17.8 (16.6)
Smoking intensity (cig./day)	6 (8.9)	14.4 (9.4)	5.5 (5.8)	10.3 (5.5)	7.7 (8.6)
Comprehensive Smoking Index (CSI)	0.5 (0.7)	1.4 (0.8)	0.7 (0.8)	1.4 (0.7)	0.8 (0.8)
Time to diagnosis (years)		7.2 (3.7)		3.9 (2.0)	5.9 (3.5)
*Centre (EPIC-Italy)*					
Florence	92 (18%)	63 (34%)			
Naples	11 (2%)	3 (2%)			
Ragusa	29 (6%)	14 (8%)			
Turin	246 (48%)	60 (32%)			
Varese	134 (26%)	45 (24%)			

### Associations with future lung cancer risk

**Fig. 1:**
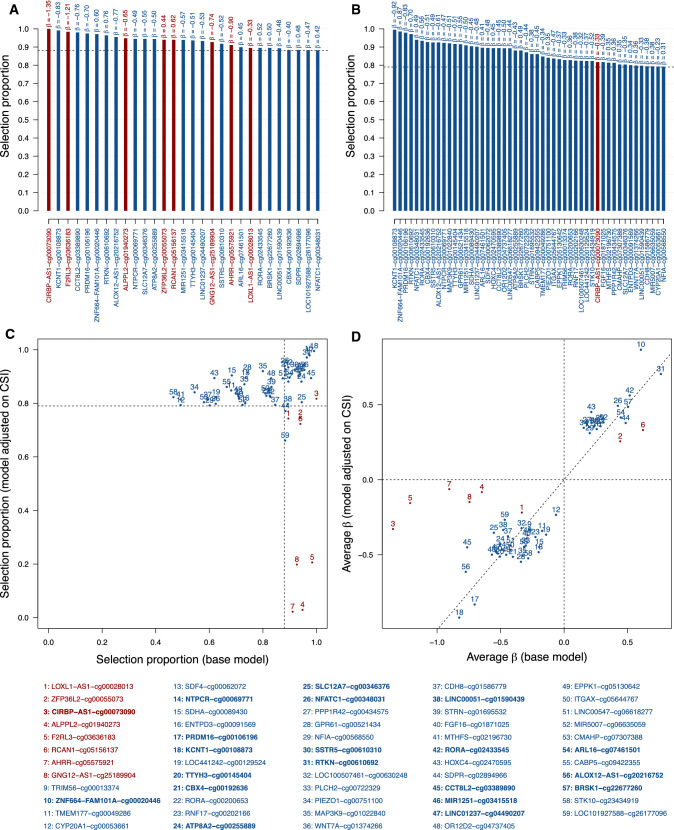
Stability selection models exploring the joint associations between CpG sites and the future risk of lung cancer. Selection proportions of stably selected CpG sites are derived from LASSO-penalised logistic models for the risk of lung cancer including all N = 443,150 CpG sites as predictors and adjusted for age, sex (**A**) and CSI (**B**). Comparison of selection proportions (**C**) or β-coefficients (**D**) from the base versus CSI-adjusted models for CpG sites that are stably selected in at least one of these two models. The list of stably selected CpG sites is reported at the bottom, with overlapping signals in bold. CpG sites related to smoking at a Bonferroni-corrected significance level ensuring a family-wise error rate below 0.05 are presented in red, and sites unrelated to smoking in blue

A total of N = 29 CpG sites (including N = 8 smoking-related and N = 21 smoking-unrelated CpG sites) were stably selected in the base model (Fig. 1A, Supplementary Table 1). In the CSI-adjusted model, N = 50 CpG sites (1 smoking-related and 49 smoking unrelated sites) were selected. Of these, N = 1 smoking-related (*CIRBP−AS1*−cg00073090), and N = 19 smoking-unrelated CpG sites were selected across both models (Fig. 1B, Supplementary Table 2). We observed lower selection proportions and effect sizes for the N = 7 smoking-related sites selected in the base model *only* (Fig. 1C, D). Despite stable inclusion in both models, *CIRBP−AS1*−cg00073090 had a lower effect size in the CSI-adjusted model (average β coefficient of − 1.35 in the base model and − 0.33 in the adjusted model). Selection proportions and effect size estimates remained generally unchanged upon adjustment for CSI for the (N = 19) smoking-unrelated CpG sites selected in both models. An additional set of N = 30 smoking-unrelated CpG sites was stably selected in the CSI-adjusted model (out of a total of 50 selected CpG sites). Although these were not stably selected in the base model, they showed selection proportions above 0.46 and comparable average β coefficients (results not shown).

Effect sizes obtained from our recalibrated models with and without adjustment for estimated white blood cell proportions showed very good consistency in both models unadjusted (Supplementary Fig. 2A) and adjusted for CSI (Supplementary Fig. 2B), hence suggesting limited confounding by cell type differentials.

### ROC analyses: quantifying the disease relevant information

**Fig. 2:**
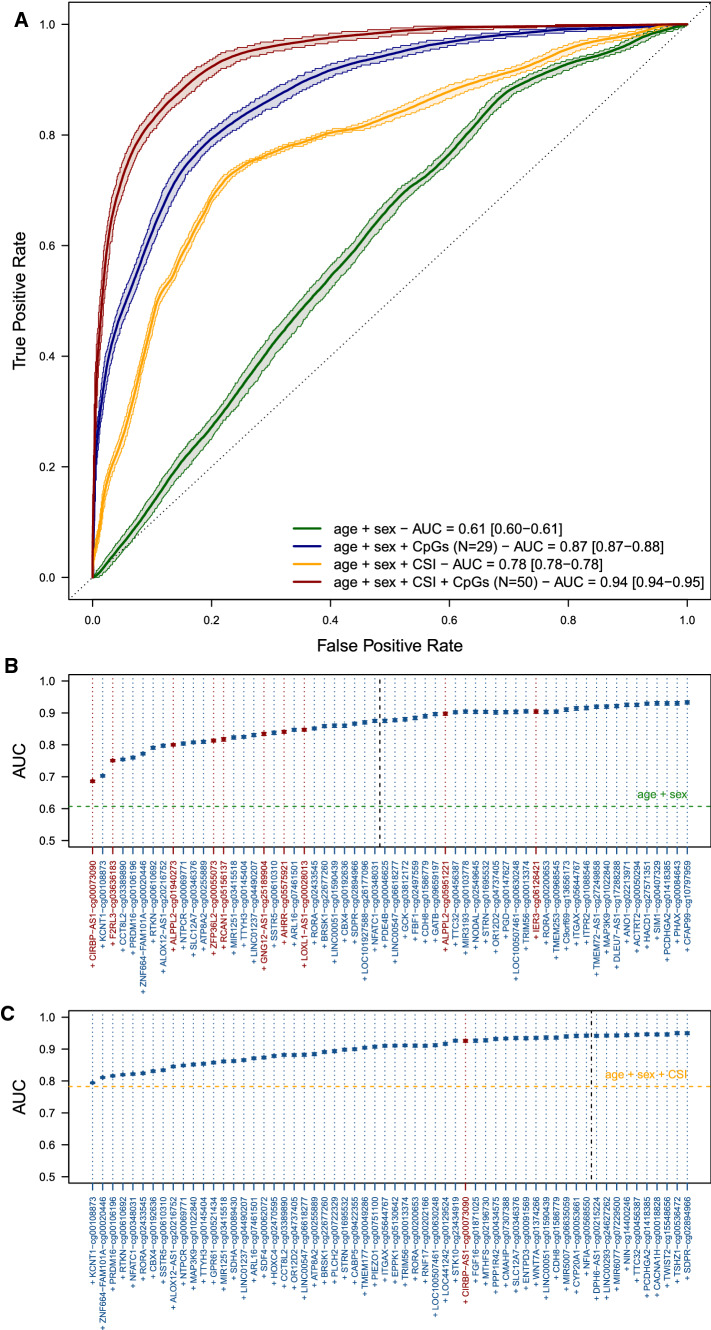
Receiver Operating Characteristic curve for lung cancer prediction. Mean and 5th–95th percentiles of the Area Under the Curve (AUC) were calculated across the 1000 recalibrated models including (i) age and sex (green), (ii) age, sex and stably selected CpG sites from the base model (dark blue), (iii) age, sex and CSI (orange), and (iv) age, sex, CSI and the stably selected CpG sites from the adjusted model (dark red) (**A**). Mean and 5th–95th percentiles of the AUC are reported for models sequentially including the first 50 CpG sites by order of selection proportion in the base (**B**) and adjusted (**C**) models. Calibrated stability selection models are indicated by a black dashed vertical line. CpG sites related to smoking at a Bonferroni-corrected significance level ensuring a family-wise error rate below 0.05 are presented in red, and sites unrelated to smoking are presented in blue

ROC analyses performed in the 1000 testing sets each including 20% of the full population yielded a mean AUC of 0.87 (5th–95th percentiles 0.87–0.88) for the model including age, sex and the N = 29 stably selected CpG sites from the base model, yielding an increase in average AUC of 0.26 over models including age and sex only and an increase of 0.09 over the model including age, sex and CSI (Fig. 2A), which had an AUC of 0.78 (5th–95th percentiles 0.77–0.78). The addition of each stably selected CpG site only incrementally improved the AUC, with the largest contributions from smoking-related CpGs, namely *CIRBP−AS1*−cg00073090 and *F2RL3*−cg03636183 (Fig. 2B: ΔAUC > 4%). The best performing model was that including CSI *and* the 50 CpG sites selected in the CSI-adjusted model. It yielded and AUC of 0.94 (5th–95th percentiles 0.94–0.95), representing an increase in mean AUC of 0.16 compared to that of the model including age, sex and CSI, and an AUC increase of 0.07 compared to that of the model including age, sex and all (N = 29) stably selected CpG sites in the base model (Fig. 2A). For both models, we observed a limited increase in AUC by including CpG sites with selection proportion below the calibrated threshold (Fig. 2B, C).

### Conditional Independence Network

**Fig. 3:**
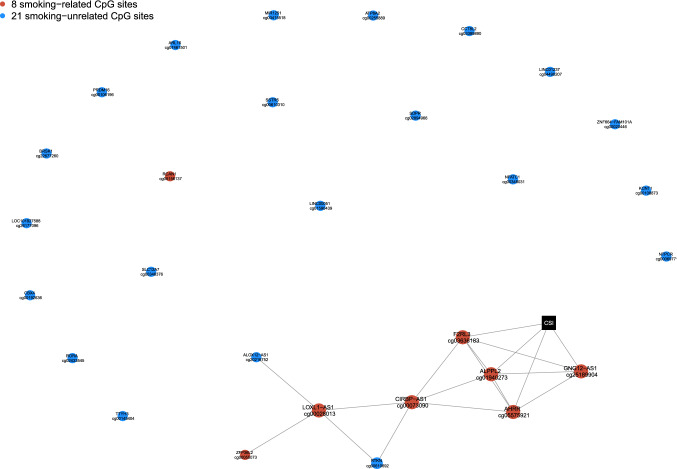
Conditional independence network including stably selected CpGs identified in relation to lung cancer in the base LASSO model (N = 29 stably selected CpG markers) and CSI (black square). CpG sites related to smoking at a Bonferroni-corrected significance level ensuring a family-wise error rate below 0.05 are presented in red, and sites unrelated to smoking are presented in blue

The stability-enhanced conditional independence network of CSI and the 29 CpG sites selected in the base model (Fig. 3) included (N = 20) edges between a set of (N = 9) inter-connected nodes (module), mostly constituted of smoking-related CpG sites (N = 7). Of these, four were directly related to CSI (*AHRR*-cg05575921, *ALPP2*-cg01940273, *F2RL3*-cg03636183 and *GNG12-AS1-cg25189904*). The remaining stably selected CpG sites (1 smoking-related, 19 smoking-unrelated) were not connected to any other node, suggesting their independence and weaker links to smoking.

## Discussion

We performed variable selection from full-resolution DNA-methylation profiles using stability selection approaches. Of the 443,150 assayed CpG sites, we identified a sparse subset of 29 CpG sites in the base LASSO model, jointly associated with the future risk of developing lung cancer. These included (N = 8) sites which were previously reported to associate with smoking and (N = 21) CpG sites, which were not.

Our conditional independence network shows that 7 of the 8 smoking-related CpG sites are inter-connected and (directly or indirectly) linked to CSI, while the (N = 21) CpG sites not reported to be associated with smoking appeared more independent and not related to smoking in our data. This lends plausibility to our CpG sites classification and suggests that the smoking-unrelated CpG sites we found associated with lung cancer risk may each capture non-redundant and complementary disease-relevant information.

Among the selected smoking-related CpG sites, three were previously identified as being associated with lung cancer risk (*AHRR*-cg0557921, *F2RL3-*cg03636183 and *ALPPL2-*cg01940273) [11, 27, 28]. Conversely, *CIRBP-AS1-*cg0073090 was not previously associated to lung cancer risk, although recent work identified *CIRBP* as hypomethylated in cases developing occult lymph node metastases among non-small cell lung cancer cases [29]. A genome wide analysis conducted with multivariate analysis also identified *CIRBP* as an important prognostic gene for non-small cell carcinoma [30].

Models adjusted for CSI selected only one CpG found to be associated with smoking (CIRBP-AS1-cg00073090), possibly indicating that this site may reflect smoking exposure that is not fully captured by CSI. In a subset of our study population, we found that methylation levels at CIRBP-AS1-cg00073090 were only nominally associated with childhood exposure to tobacco smoke and that this association did not survive correction for multiple testing and adjustment for CSI. This suggests that early-life exposure cannot fully explain the association linking methylation level at CIRBP-AS1-cg00073090 and future risk of lung cancer. However, short-term, environmental/occupational, second-hand exposure to tobacco smoke can potentially confound this association.

The remaining lung cancer-relevant information is captured by a set of (N = 49) smoking-unrelated CpG sites, potentially reflecting a large range of biological pathways. The fact that we highlighted novel, smoking-unrelated CpG sites upon accounting for the effect of CSI, tends to be in line with former investigations, which also capture weaker effect-size and more heterogeneous signals when compared to the generally conserved smoking-related epigenetic signature (i.e. AHRR-cg05575921, F2RL3-cg03636183) [15]. Among these 49 smoking-unrelated markers, 33 were found hypomethylated and 17 hypermethylated in lung cancer cases, and our discussion will be restricted to those presenting stronger effects and a high selection proportion (|β|> 0.5, selection proportion > 0.9, N = 14). Following adjustment for CSI, the most frequently selected CpG site was KCNT1-cg00108873 which was hypomethylated in lung cancer cases. KCNT1 is a protein coding gene involved in the intracellular potassium activated channel activity. It has, to our knowledge never been associated to lung cancer risk, although recent work on gene expression identified low expression of KCNT1 consistently in four cancers, including lung [31]. Additional smoking-unrelated CpG sites selected in the CSI-adjusted model included PRDM16-cg00106196, CBX4-cg00192636, SSTR5-cg00610310, ALOX12-AS1-cg20216752, NTPCR-cg00069771, MAP3K9-cg01022840, TTYH3-cg00145404, GPR61-cg00521434, and MIR1251-cg03415518, which were all hypomethylated (β < -0.5), whereas ZNF664-FAM101A-cg0020446, RTKN-cg00610692, NFATC1-cg00348031, and RORA-cg02433545 were hypermethylated (β > 0.5). We found that PRDM16 encodes for a zinc transcription factor which controls the development of brown fat cells. While its role in lung cancer has been poorly investigated, recent work has shown that it could potentially be an interesting therapeutic target for lung adenocarcinomas [32]. Further, we found that CBX4 has been specifically related with lung cancer development, acting as an oncogene that enhances cell proliferation and promotes cancer cell migration [33]. SSTR5 encodes for the somatostatin receptor type 5, which is involved in signalling to alter hormone secretion, increase apoptosis, and decrease cellular proliferation. In concordance with our findings, differential expression of SSTR5 has been observed in neuroendocrine lung cancer cases in respect to controls [34]. TTHY3 is a gene coding for an intracellular calcium activated chloride channel activity. No observations linking TTHY3 to lung cancer risk were found, although its differential expression has been linked to poor clinical outcomes in cases of gastric cancer [35]. We have also found that ALOX12 (arachidonate 12-lipoxygenase) was associated with lung cancer risk. This gene has not been specifically related to lung cancer risk, but former studies have reported (i) that hypomethylation at one of its CpG site was associated with inflammation of the airways, and (ii) that ALOX12 expression was upregulated in in breast cancer tissue [36–38]. We could not find evidence for an association between ZNF664-FAM101A and NTPCR genes and lung cancer, although ZNF664-FAM101 expression was found to be up-regulated in gastric cancer tissue [39]. Among the CpG sites we identified, some were located in intragenic regions of the RTKN1, GPR61, MAP3K9, and CABP5 genes, whose functions include cell signalling, cell cycle control, and cell growth regulation [40]. Although only MAP3K9 has been specifically related with lung cancer, acting as a cell proliferation promoting factor (oncogene) [41], previous research has reported that an increased expression of RTKN1 was observed in multiple cancer types, including lung cancer [42, 43], whereas CABP5 and GPR61 were previously related with increased RNA expression in human gastric cancer tissue, and aberrant methylation patterns resulting from air pollution, respectively [44, 45]. Exploring the roles of NFATC1, RORA, and MIR1251, we found that all three genes were implicated in lung cancer, with NFATC1 being an oncogene, RORA being a key circadian clock regulator in non-small cell lung carcinoma, and MIR1251 encoding for non-coding RNA which promotes cell migration and invasion by lung cancer cells [46–48].

Finally, we explored the role of the 36 remaining markers by applying a gene ontology approach, in order to highlight the general processes in which the identified genes may be involved [49]. Seven major pathways were identified, including FGF, Integrin, Wnt, and Cadherin signalling pathways, as well as Angiogenesis, Gonadotropin-releasing hormone receptor, and the Alzheimer disease-presenilin pathway, with all seven pathways being implicated in lung cancer [50–56].

## Strengths and limitations

To our knowledge, this is the first study to investigate associations between epigenome-wide methylation profiles and lung cancer risk by applying stability selection. This approach has allowed us to highlight two sets of CpG sites that were explanatory of lung cancer: those related and those unrelated to smoking.

Our study also has several limitations. First, we examined DNA methylation from blood, and not from more proximal tissues such as the lung epithelium, buccal cells, or sputum, which may represent a challenge regarding the interpretability of the identified CpG sites from the pathophysiological perspective. Second, we used a classification of CpGs according to their associations with smoking in previous large meta-analyses [20]. Our classification relies on marginal associations, which may result in potential misclassification of CpG sites and may overlook potential interactions between smoking-related CpG sites and those initially classified as smoking-unrelated [20]. Nevertheless, our conditional independence network seems to support this classification. In addition, exposure to passive smoking has not been taken into account in our analyses, hence, some of the CpG sites we refer to as being smoking unrelated CpG sites may still be related to other routes of exposure to tobacco smoke (e.g. second-hand smoking, environmental exposure, early-life exposure). Further, even though we observed a high consistency across smoking-unrelated CpGs found in the base and CSI-adjusted models (N = 19 smoking-unrelated CpG sites selected across both models), an additional (N = 30) smoking-unrelated CpG sites were selected in the CSI-adjusted model. These consist of several (uncorrelated) CpG sites capturing complex (and inherently multidimensional) molecular pathways, other than those related to smoking, involved in lung carcinogenesis. Another limitation of our work is that women are largely over-represented in our study. Although sex-stratified analyses showed similar predictive performances of our selected CpG sites in men and women separately (results not shown), additional work would be warranted to assess the potential sex bias that could have been introduced as well as the effect of sex-specific smoking patterns, by replicating our results in other studies. Although our results are based on the comparison of prospective cases to controls, reverse causation, whereby some of the differentially methylated sites we identify would relate to developing and yet not diagnosed cancer rather than to mechanisms involved in carcinogenesis cannot be completely ruled out. However, while excluding (27%) lung cancer cases that were diagnosed less than 3 years after blood sampling from our analyses, most of the CpG sites we identified in the full data set were selected and those which were not had selection proportion > 50%. This lends plausibility to our findings, but formal assessment of reverse causation would entail the longitudinal analysis of repeated measurement of DNA methylation signals and smoking metrics across the life-course. Overall, larger studies would be needed to investigate the validity of our findings at different time to diagnosis, and across main histological subtypes. Finally, our CpG methylation data was measured using the Illumina HM450 methylation array in both EPIC-Italy and NOWAC, it yields a more limited coverage than more recent assays, such as the Infinium Methylation EPIC, which includes 850′000 CpG sites [57].

## Conclusion

In conclusion, our study uses novel penalised regression coupled stability selection approaches to generate new hypotheses on potential mechanisms other than those related to smoking that are involved in lung carcinogenesis. We highlighted multiple CpG markers that are strongly and consistently associated with future risk of lung cancer, with a distinct subdivision between few, correlated, CpG sites that have been previously associated with smoking, and multiple, smoking unrelated CpG sites. Based on this classification, which was supported by our network analyses, we found that two smoking-related CpG sites improved the discriminatory performances of the model, over and above that of CSI. These may therefore capture lung cancer relevant effects of life-course exposure to tobacco smoke. We also selected a set of 49 CpG sites not directly linked to smoking, that were complementarily explanatory of lung cancer risk. If validated in independent data, these may prove instrumental in understanding biological mechanisms involved in lung carcinogenesis that are not directly linked to smoking. Overall, our findings identify sets of differentially methylated sites that are jointly explanatory of future lung cancer risk, these may provide leads into the mechanisms involved in lung cancer development (with and without the implication of smoking) and may prove useful for better early identification of patients at higher risk of lung cancer.

## Supplementary Information

Below is the link to the electronic supplementary material.

Supplementary file1 Figure 1: Flow-chart of included participants and CpG markers in EPIC-Italy and NOWAC samples.

Supplementary file2 Figure 2: Recalibrated regression coefficients from the base model (A) or CSI-adjusted model (B) without (X axis) and with (Y axis) adjustment for estimated white blood cell proportions of Monocytes, B cells, CD4+ T cells, Natural Killers, CD8+ T cells, and Neutrophil.

Supplementary file3 Table 1: Results from the base stability selection model. Models are adjusted on age and sex (unpenalised). Stably selected CpG sites are reported, along with their selection proportions and their average β-coefficient conditionally on selection across the 1000 subsamples. Table 2: Results from the adjusted stability selection model. Models are adjusted on age, sex and CSI (unpenalised). Stably selected CpG sites are reported, along with their selection proportions and their average β-coefficient conditionally on selection across the 1000 subsamples.

## Data Availability

The EPIC and NOWAC data cannot be shared publicly because of local and national ethical and security policy. Data access for researchers will be conditional on adherence to both the data access procedures of the Norwegian Women and Cancer Cohort and the UiT The Arctic University of Norway (contact via Torkjel Sandanger, torkjel.sandanger@uit.no, Tonje Braaten tonje.braaten@uit.no, and Arne Bastian Wiik, arne.b.wiik@uit.no) for NOWAC in addition to the local ethical committee. Access to EPIC data can be request upon application and acceptance from the project steering committee (epicadmin@imperial.ac.uk), data sharing will also be conditional to ethic approval and compliance to GDPR regulations.

## Abbreviations

AUC: Area under the curve
CSI: Comprehensive smoking index
EPIC: European prospective investigation into cancer and nutrition
LASSO: Least absolute shrinkage and selection operator
NOWAC: Norwegian women and cancer study
ROC: Receiver operating characteristics

## Code availability

The computer code used to support the findings of this study are available on GitHub, at https://github.com/barbarabodinier/Epigenetic_mechanisms_lung_cancer
