# Supplementary figures and images for: Epigenetic mechanisms of lung carcinogenesis involve differentially methylated CpG sites beyond those associated with smoking
Source: Eur J Epidemiol. 2022 May 20;37(6):629–40. doi: 10.1007/s10654-022-00877-2 (PMC9288379; doi:10.1007/s10654-022-00877-2)

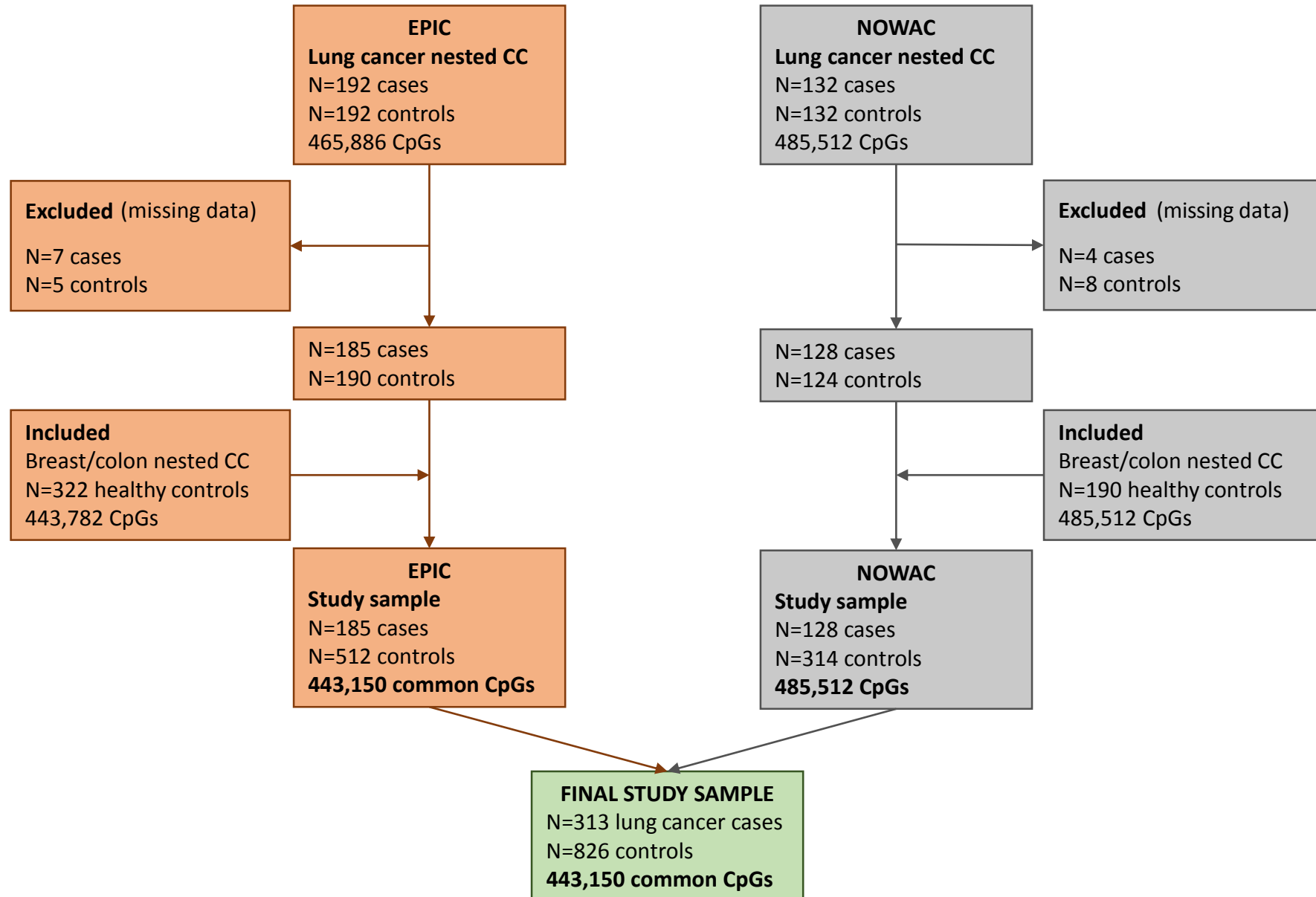

Supplement: Supplementary file 1 — Supplementary file1 Figure 1: Flow-chart of included participants and CpG markers in EPIC-Italy and NOWAC samples. [file 10654_2022_877_MOESM1_ESM.pdf]

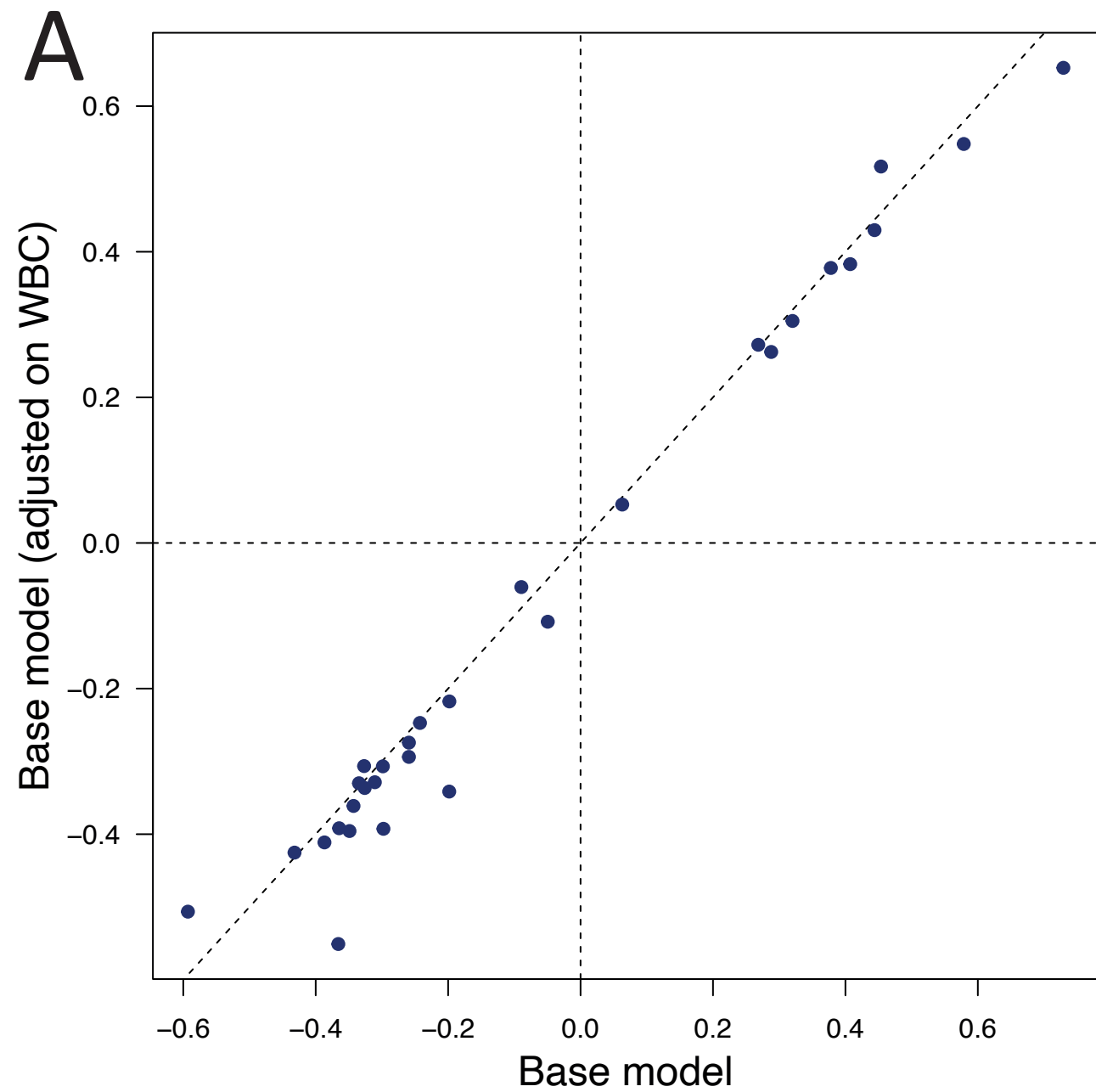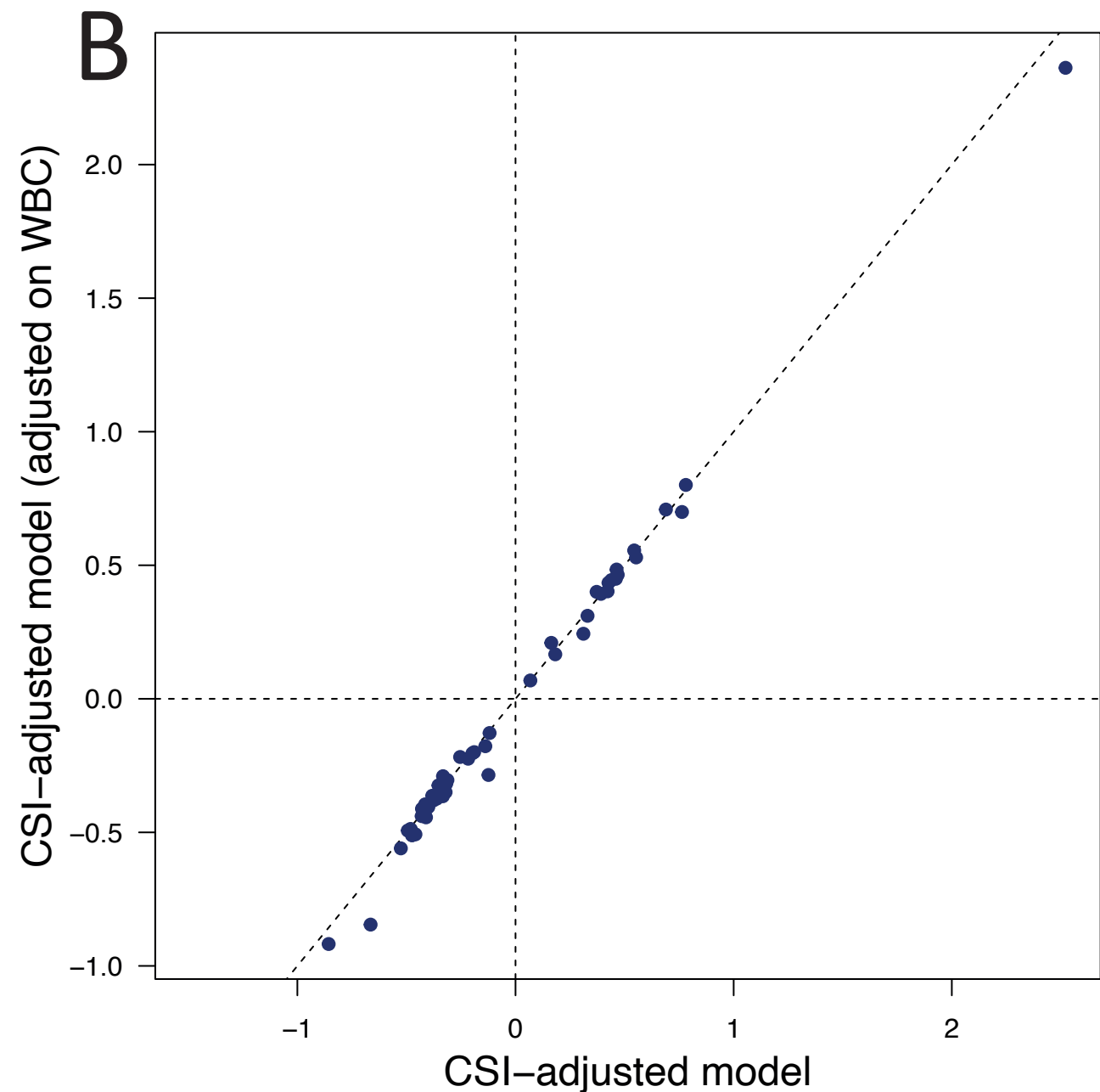

Supplement: Supplementary file 2 — Supplementary file2 Figure 2: Recalibrated regression coefficients from the base model (A) or CSI-adjusted model (B) without (X axis) and with (Y axis) adjustment for estimated white blood cell proportions of Monocytes, B cells, CD4+ T cells, Natural Killers, CD8+ T cells, and Neutrophil. [file 10654_2022_877_MOESM2_ESM.pdf]
